# Effects of exercise-based interventions on gluteal tendinopathy. Systematic review with meta-analysis

**DOI:** 10.1038/s41598-024-53283-x

**Published:** 2024-02-09

**Authors:** Thaisy Thuany Patricio Cordeiro, Emannuel Alcides Bezerra Rocha, Rodrigo Scattone Silva

**Affiliations:** 1 Postgraduate Program in Rehabilitation Sciences (PPGCREAB), Health Sciences College of Trairi, Federal University of Rio Grande do Norte (UFRN/FACISA), Santa Cruz, RN Brazil; 2 Brazilian Tendinopathy and Sports Injuries Research Group (BRATSI), Federal University of Rio Grande do Norte, Santa Cruz, RN Brazil; 3 Postgraduate Program in Physical Therapy (PPGFIS), Federal University of Rio Grande do Norte (UFRN), Natal, RN Brazil

**Keywords:** Diseases, Health care

## Abstract

The objective of this review was to evaluate the effect of exercise on pain intensity, function, and quality of life in individuals with gluteal tendinopathy. Searches were carried out in PUBMED, EMBASE, CINAHL, Cochrane Library, and PEDro databases. Randomized or quasi-randomized controlled trials were included. Five studies met the eligibility criteria, comparing exercise-based interventions with minimal interventions and/or corticosteroid injections. Three studies, involving 383 participants, were included in the quantitative analysis. Meta-analyses showed that exercise is superior to minimal intervention for function in short-term [mean difference (MD) = 10.24; 95% confidence interval (95%CI) = 5.98, 14.50) and long-term (MD = 6.54; 95%CI = 1.88, 11.21]). However, no difference was observed for quality of life in the short [standardized mean difference (SMD) = 0.33; 95%CI = −0.29, 0.94] and long-term (SMD = 0.11; 95%CI = −0.16, 0.37). The effect of exercise was no different from that of corticosteroid injections for pain intensity in the short (MD = 1.25; 95%CI = −3.56, 6.05) and long-term (MD = −1.37; 95%CI = −3.72, 0.98]). In conclusion, exercise is superior to minimal interventions for function in the short- and long-term in individuals with gluteal tendinopathy. Exercise and corticosteroid injections had similar effects on pain intensity, however, exercise showed a higher treatment success rate when compared to corticosteroid injections in this population. The GRADE analysis revealed that the certainty of the evidence ranges from low to very low, therefore, large high-quality randomized controlled trials are recommended.

PROSPERO registration number: CRD42021242853.

## Introduction

Gluteal tendinopathy is the most prevalent of all lower limb tendinopathies^1^ and it is considered the most common cause of lateral hip pain^1–3^. Common synonyms for this condition include greater trochanteric pain syndrome and trochanteric bursitis^4–6^. However, as dysfunction of the gluteus medius and minimus tendons has been considered the primary cause of pain in these individuals, the term 'gluteal tendinopathy' has been recommended to designate lateral hip pain of insidious onset^7^.

It is a common condition in adults, both sedentary and athletes, with an annual incidence of 1.8 per 1000 individuals^8^, and a global prevalence of 20.2%^9^. It affects individuals with an age range of 15–87 years and an average age of 54 to 63 years. Women are typically more affected when compared to men^6,9,10^. Recently, it has been demonstrated that gluteal tendinopathy causes disability and reduced quality of life of an intensity equivalent to that observed in the late stages of hip osteoarthritis^11^. This fact reflects on the individual's functionality levels and can result in a reduction in the number of hours worked full-time^11^.

Individuals with gluteal tendinopathy often report exacerbated symptoms during activities of daily living, such as walking, climbing stairs, sitting, and getting up from a chair and lying on the affected side^11^. Symptoms can persist for up to 5 years after the onset in 29% of cases^8^. Regarding symptoms, gluteal tendinopathy is characterized by pain with an insidious, that manifests itself chronically, intermittently or continuously, the proximal lateral aspect of the hip, which may radiate to the distal thigh^7,11,12^. Pain and tenderness on palpation of the greater trochanter are represented as the main diagnostic criteria for gluteal tendinopathy^12^.

Due to the high prevalence and disabling nature of gluteal tendinopathy, the effectiveness of conservative interventions for the treatment of this condition has been investigated in some studies^13,14^. Among conservative interventions, exercise has been shown to be an effective therapy for the treatment of tendinopathies in general^15–18^, being considered the preferred option for managing these conditions^19^. Several studies have shown that exercise-based interventions have significant results in terms of reducing pain and improving function in individuals with lower limb tendinopathy^20–23^, such as patellar and Achilles tendinopathy. However, to the best of our knowledge, the effects of exercise-based interventions for the treatment of gluteal tendinopathy are not completely understood and need to be systematically reviewed and analyzed.

Therefore, the aim of this review was to evaluate the effects of exercise-based interventions on pain intensity, function, global perception of change, and quality of life in individuals with gluteal tendinopathy. Secondary objectives included evaluating the effects of exercise-based interventions on pain catastrophizing, strength, range of motion, biomechanical variables, and activity participation, as well as the occurrence of adverse events.

## Methods

This systematic review was conducted following the recommendations of the Cochrane Collaboration and is being presented in accordance with the Preferred Reporting Items for Systematic Reviews and Meta-Analyses (PRISMA) guidelines^24^. The review protocol was prospectively registered with PROSPERO (CRD42021242853).

### Selection criteria for included studies

#### Study design

Systematic review with meta-analysis which included randomized and quasi-randomized controlled trials. Studies with other types of study designs, such as case series and case reports, were excluded.

#### Population

Studies that evaluated patients diagnosed with gluteal tendinopathy, greater trochanteric pain syndrome, trochanteric bursitis or nonspecific lateral hip pain^7,25^, diagnosed clinically, with or without abnormal imaging findings were included. For inclusion, participants had to be over 18 years old, of both sexes, with any level of physical activity. Studies involving patients diagnosed with hip osteoarthritis, femoroacetabular impingement, partial or complete rupture of the gluteal tendons, labrum injuries, avascular necrosis of the femoral head, hip fractures, peripheral nerve dysfunctions (such as lateral femoral cutaneous nerve entrapment), rheumatological diseases and/or tumors were excluded.

#### Interventions and comparisons

Studies that carried out interventions based on progressive load resistance exercises, with concentric, eccentric, and/or isometric contractions in at least one of the groups were included. Resistance training could have been carried out in isolation or combined with other treatments, supervised by health professionals or unsupervised, using any progression methods. Studies were considered eligible if they compared an exercise group to: other exercise-based interventions, placebo/sham interventions, no intervention (such as 'wait and see' approach), education, electrothermophototherapeutic interventions (modalities such as extracorporeal shockwave therapy, ultrasound, etc.), invasive interventions (such as corticosteroid or platelet rich plasma injections), or any other type of conservative or surgical intervention.

#### Outcomes

The primary outcomes were: pain intensity [Visual Analogue Scale (VAS) and Numerical Pain Rating Scale (NPRS)]; function [Victorian Institute of Sport Assessment-Gluteal tendons (VISA-G), Oxford Hip Score (OHS) and Lateral Hip Pain Questionnaire (LHPQ)]; perception of change [Global Rating of Change Scale (GROC)] and quality of life [Assessment of Quality of Life (AQoL) and Quality of Life Questionnaire (EQ-5D)].

Secondary outcomes were: participation in activities [Active Australia Survey (AAS) and International Physical Activity Questionnaire-Short Form (IPAQ-SF)]; strength (dynamometry); biomechanical variables (kinematics and kinetics of the pelvis, trunk and lower limbs); and pain catastrophizing [Pain Catastrophizing Scale (PCS)]. Studies that included assessments of any of these outcomes using the aforementioned instruments, or any validated and reliable instrument, were included.

### Research methods for identifying studies

#### Electronic search

The databases Cochrane Central Register of Controlled Trials (CENTRAL, Cochrane Library), Medical Literature Analysis and Retrieval System Online (MEDLINE, via PubMed), Cumulative Index to Nursing and Allied Health Literature (CINAHL, via EBSCO HOST), Excerpta Medica dataBASE (Embase), and Physiotherapy Evidence Database (PEDro) were searched on March 30, 2021 and the searches were updated on March 3, 2022. No language or date restrictions were applied. Search strategies were developed using relevant keywords, which were combined with Boolean terms (see Suppl. Appendix A for complete search strategy).

### Data collection and analysis

#### Study selection

All publications identified in the databases were exported to the Rayyan software (Rayyan QCRI/web app) to remove duplicates and for the study eligibility assessment process. Two reviewers (TTPC and EABR) independently evaluated all articles, initially by titles and abstracts. After this stage, the full texts of potentially relevant studies were evaluated by the same two reviewers. In addition to the electronic search, a manual search of reference lists of the included studies and of review articles was performed to identify other potentially relevant studies. All included articles were discussed to minimize the risk of bias, as recommended by the Cochrane Manual for Systematic Reviews of Interventions^26^. Disagreements regarding the eligibility of studies were discussed and resolved by consensus and, if disagreement persisted, a third reviewer was contacted (RSS). In cases where eligibility criteria were unclear, authors were contacted by email.

#### Data extraction

Data extraction was performed independently by two reviewers (TTPC and EABR)^26^. When consensus could not be reached, the decision was determined by a third reviewer (RSS). Data were extracted using a standardized form, prepared following the instructions of the data collection form for intervention reviews, developed by Cochrane^27^.

The following items were extracted: objective and study design, sample size, inclusion and exclusion criteria, follow-up time, randomization method, allocation concealment, blinding and other strategies to minimize the risk of bias; patient demographic data; intervention details, such as treatment and session duration, session frequency, dosage of interventions and co-interventions; outcomes of interest, such as pain, function, perception of change, quality of life, strength, participation in activities, pain catastrophizing, and adverse events, including the times at which they were measured; number of participants in each group, calculation of sample size and power. Data regarding differences between the post-intervention groups in the short, medium, and long term were extracted. Categorical and continuous outcomes, confidence intervals, imputation of missing data, and missing data at each time point were considered. In cases of missing data, the authors were contacted by email, with a request to share the results.

#### Risk of bias

The Physiotherapy Evidence Based Database (PEDro) scale was used to evaluate the methodological quality of the studies^28,29^. The evaluation of the included studies was also carried out independently by two reviewers (TTPC and EABR)^26^, and when consensus could not be reached, the decision was determined by a third evaluator (RSS). At the end of the evaluations, the scores were compared with the results from the PEDro database and there was no divergence between the evaluated scores and the scores obtained from the PEDro database. In terms of study methodological quality, it has been suggested that studies with a score < 4 are considered poor, between 4 and 5 are considered fair, 6 to 8 are considered good, and between 9 and 10 are considered excellent^30,31^.

#### Data analysis

Whenever possible, data were grouped, and meta-analysis was performed using the random effects model and presented in a forest-plot graph. Results were displayed as mean difference (MD) and 95% confidence intervals (95%CI) when studies used the same scales. Otherwise, effects were calculated using standardized mean difference (SMD) and 95% CI. Chi^2^ and I^2^ statistics were used to measure heterogeneity between the analyzes. All analyzes were performed using Review Manager software, version 5.4. For effect measures, short-term effects were considered when follow-up was ≤ 12 weeks and long-term effects were considered for follow-up ≥ 26 weeks.

Finally, the assessment of the strength of the evidence was carried out using the Grading of Recommendations Assessment (GRADE) approach^32^ through the GRADE PRO software^33^. As this was a systematic review of randomized clinical trials, downgrade criteria were used. Downgrade for each of the following items was considered: risk of bias, when > 25% of participants were from studies with high risk of bias (PEDro score < 6/10)^34^; imprecision, when the total sample was < 400^35^; serious inconsistency of results, when the I^2^ statistic was greater than 50% or when grouping was not possible^26^; and indirect evidence, when there were comparisons between different populations and interventions^36^. Publication bias could not be assessed as fewer than ten studies were included^35^. After evaluating all domains, the certainty of the evidence was classified into one of the following levels^36^: high certainty (very confident that the true effect is close to the estimated effect); moderate certainty (moderately confident that the true effect is likely to be close to the effect estimate, but there is a possibility that it will be substantially different); low certainty (confidence in the effect estimate is limited and the true effect may be substantially different from the effect estimate); very low certainty (little confidence in the effect estimate and the true effect is likely to be substantially different from the effect estimate)^32^.

## Results

**Figure 1:**
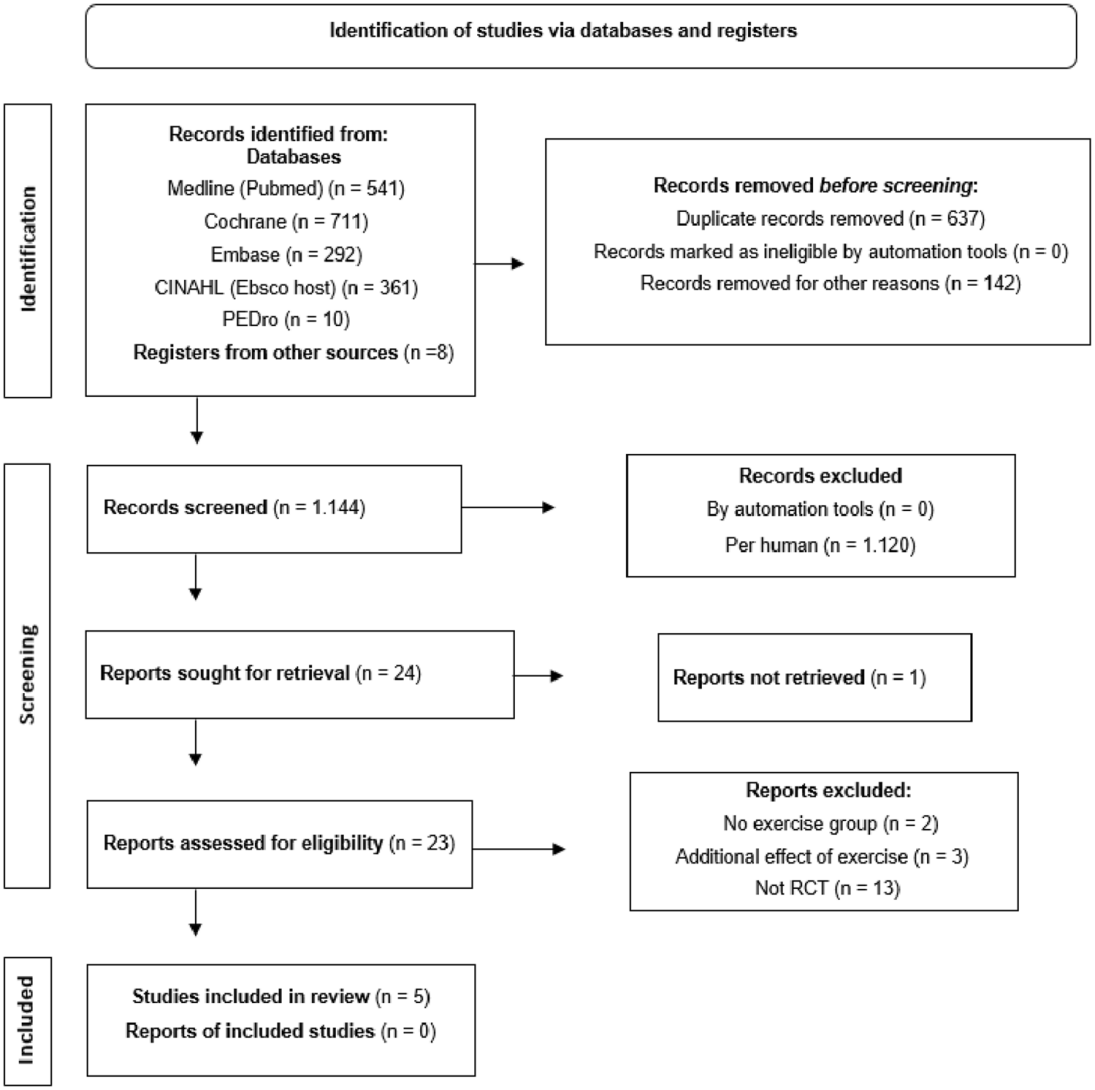
Flowchart of review studies following PRISMA recommendations. *RCT* randomized clinical trial, *PRISMA* Preferred Reporting Items for Systematic Reviews and Meta-Analyses.

The electronic search identified 1,923 studies. Twenty-three studies had the full text read and 18 were excluded for not meeting the eligibility criteria. Detailed reasons for exclusion are described in Fig. 1. Finally, five studies were included for analysis. Qualitative results regarding samples, interventions, outcomes, and adverse events are described in Table 1. Data regarding the methodological characteristics of the included studies are presented in Table 2.

**Table 1:** Samples, intervention characteristics, outcome measures, and adverse effects of the included studies.

Reference	Sample, mean age, sex F (%)	Interventions	Duration of treatment	Outcome measures (Follow-up)	Co-intervention(s)	Adverse events
Cowan et al. 2022	n = 132 Mean age 61.1 F = 132 (100%)	Isometric and kinetic chain exercises (n = 63): 10–15 min, 2×/day, involving isometric exercises for the gluteus medius and minimus, and strengthening of the kinetic chain (quadriceps and calf). Participants were instructed to complete 2–4 × of 5–15 repetitions, depending on the participants' level of function and stage Sham exercises (n = 69): sitting exercises not aimed at producing therapeutic loading of the gluteal tendons or strengthening the kinetic chain. Dosage for all exercises the same as the dynamic exercises of the other group	Exercise protocols: 12 weeks MHT and placebo: 12 weeks	1.VISA-G 2. OHS 3. GROC 4. AQoL 5. HOOS (pain, symptoms, ADL, sport, and QOL subscale) (12 and 52 weeks)	MHT: transdermal hormone cream composed of estradiol (50 mcg) and norethindrone/norethisterone acetate (140 mcg; a synthetic progestogen). Application of 1 mL of cream to the inner forearm once a day Placebo: an aqueous transdermal cream, but without active ingredients. Application of 1 ml of cream to the inner forearm once a day Education: verbal education and written information on how to avoid gluteal tendon compression and load management during ADL and exercise	Mild skin reaction. Placebo (n = 1)
Clifford et al. 2019	n = 30 Mean age 59.3 F = 27 (90%)	Isometric exercises (n = 15): hip abduction at 30° with knee extension in side-lying, holding for 30 s. 6×, with 60 s rest. Weight-bearing gluteal contraction, performing abduction/adduction, maintaining 6 s. 3 × 10 with 60 s rest between each set Isotonic exercises (n = 15): hip abduction to 30° with knee extension in side-lying. Standing hip abduction, sliding the foot on the floor, and maintaining knee extension. Both exercises: 3 × 10 with 60 s rest between each set. Repetition lasting 6 s (3 s concentric, 3 s eccentric)	12 weeks	1. VISA-G 2. NRS 0–10 for pain 3. EQ-5D-5L 4. IPAQ-SF 5. GROC 6. PCS 7. HOOS (4 and 12 weeks)	Postural education and guidance on positions that could be used to reduce pain and tendon compression during ADL	Increased hip pain Isometric (n = 1) Increased knee pain Isotonic (n = 1)
Ganderton et al. 2018	n = 94 Mean age 61.8 F = 94 (100%)	Isometric and kinetic chain exercises (n = 46): 10–15 min, 2×/day, involving isometric contraction of the gluteus medius and minimus, and strengthening of the kinetic chain (quadriceps and calf). Participants were instructed to complete 2–4 × of 5–15 repetitions, depending on the participants' level of function and stage Sham exercises (n = 48): seated exercises not aimed at producing therapeutic loading of the gluteal tendons or strengthening the kinetic chain. Dosage for all exercises the same as the dynamic exercises of the other group	12 weeks	1. VISA-G 2. OHS 3. GROC 4. AQoL 5. HOOS (subscales of pain, symptoms, ADL, sport, and QoL) 6. LHPQ (subscales of ADL and sport) (12 and 52 weeks)	Education: Educational booklet with activities to avoid and postures to reduce the compressive load on the tendon. They were instructed to apply these principles to all ADL, recreation, and sport	Increased lateral hip pain that did not improve during the 12-week intervention period Isometric and kinetic chain exercises (n = 1) Sham exercises (n = 1)
Mellor et al. 2018	n = 204 Mean age 54.8 F = 167 (82%)	Education and exercise (n = 69): functional retraining, strengthening for hip abductors and thigh muscles and dynamic control of adduction during ADL and daily home exercises (4–6 exercises daily). Gradually increased difficulty with no significant increase in pain (NRS 5/10). Detailed counseling and education on tendon care—handouts, verbal explanation, and an informative DVD Wait and see (n = 69): A session with a physical therapist to advise general tendon care and self-care and answer any questions about the condition, and information sheet on the condition and basic self-management CI (n = 66): mixture of 1 ml of celestone chronodose (betamethasone 5.7 mg/ml) or 1 mL of kenacort (triamcinolone acetonide 40 mg/ml) and 3 ml of 0.5% Bupivacaine. Application on the injection at gluteus minimus and medius tendon and trochanteric bursa	Education and exercise: 14 sessions for 8 weeks Wait and see: 1 session CI: 1 session	1. GROC 2. NRS 0–10 for pain 3. VISA-G 4. PSFS 5. EQ-5D 6. PCS 7. PHQ-9 8. AAS 9. LHPQ 10. Static painfree abductor torque 11. Abductor muscles active lag (4, 8, 12, 26, and 52 weeks)	None	No serious adverse events
Rompe et al. 2009	n = 229 Mean age 47.7 F = 162 (71%)	Training at home (n = 15): slow and progressive repetitive exercises, twice/day, 7 days a week. Piriformis stretch: 3 × 30–60 s, standing iliotibial band stretch: 3 × 30 s, straight leg raise: 3 × 10, wall squat with ball: hold position of thighs parallel to the ground 10 s, repeat 20×, gluteal strengthening: hold hip extension 5 s, 3 × 10 CI (n = 15): syringe containing 5 ml of 0.5% mepivacaine and mixed with 1 ml of prednisolone (25 mg) in the area most tender to palpation in the greater trochanter region and the rest of the medication on other painful areas ESWT (n = 15): radial shock wave device. Each session, 2000 pulses applied with a pressure of 3 bar (0.12 mJ/mm^2^). Frequency of 8 pulses/s. Area of maximum tenderness treated in a circumferential pattern, starting at the greater trochanter	Training at home: 12 weeks CI: a single injection ESWT: 3 weekly sessions	1. 6-point Likert scale: for degree of recovery (completely recovered to much worse) 2. NRS 0–10: for pain severity (1, 4, and 15 months)	For all groups, usage of analgesic medication was allowed when requested (paracetamol, 2000–4000 mg/day)	Training at home: increased pain for 1 day (n = 7) Pain increase > 1 day (n = 15) Irradiating pain (n = 5) CI: increased pain for 1 day (n = 8) Increased pain > 1 day (n = 18) Radiating pain (n = 7) Skin irritation (n = 2) Swelling (n = 7) ESWT: increased pain for 1 day (n = 8) Increase in pain > 1 day (n = 2) Radiating pain (n = 3) Skin irritation (n = 26) Swelling (n = 3) Other minor or temporary reactions (n = 1)

*F* female, *ESWT* extracorporeal shock wave therapy, *CI* corticosteroid injection, *NRS* numerical rating scale, *GROC* global rating of change, *EQ-5D* Euro Quality of Life Instrument-5D, *HOOS* Hip Disability and Osteoarthritis Outcome Score, *OHS* Oxford Hip Score, *IPAQ-SF* International Physical Activity Questionnaire Short Form; *PCS* Pain Catastrophizing Scale, *BIP* Brief Pain Inventory, *VISA-G* Victorian Institute of Sports Assessment-Gluteal, *ADL* Activity of Daily Living, *QoL* Quality of Life; *PHQ9* Patient Health Questionnaire 9, *LHPQ* Lateral Hip Pain Questionnaire, *PSFS* Patient-Specific Functional Scale, *AQol* Assessment of Quality of Life instrument; *AAS* Active Australia Survey, *MHT* transdermal hormone replacement.

**Table 2:** Description of the studies considering the methodological characteristics, inclusion, and exclusion criteria of the included studies.

References	Randomization method	Allocation concealment	Blinding method	Statistical power	Baseline comparison	Inclusion criteria	Exclusion criteria
Cowan et al. 2022	Sequence generated by computer software	Allocation performed by an independent investigator	Participants blinded to group allocation until completion of the intervention for the cream group Physiotherapists and researchers were blinded to cream allocation Physiotherapists were not blinded to exercise allocation	80%	Difference in age between Exercise/ MHT and Sham/ Placebo groups The exercise/MHT group was also heavier than the others	Being postmenopausal (> 52 weeks of menstrual interruption) and/or serum estradiol 0–120 pmol/L and FSH 0.20 IU/L. Have reproduction of lateral hip pain in three of the five pain provocation tests. Have sufficient English skills to read and complete the questionnaires and consent to the study requirements	Having received an injection in the hip area in the previous 12 weeks (PRP, autologous blood injection or CI), a history of hip trauma or surgery on the affected side, or any other musculoskeletal, neurological, metabolic, or cardiorespiratory problem. Known adverse reaction to hormone therapy
Clifford et al. 2019	Simple randomization, draqing opaque, sealed envelopes from a box	Opaque and sealed envelopes	None	None	No difference	Age > 18 years, having lateral hip pain for > 3 months, having lateral hip pain on direct palpation around the greater trochanter with pain reproduced in at least one of five pain provocation tests	Physical therapy in the last 6 months and/or received CI for lateral hip pain in the last 3 months, unable to actively abduct the affected hip in side-lying, pain reproduced with flexion, adduction, IR of the hip with concomitant hip osteoarthritis, previous surgery of hip or lumbar spine in the previous 12 months or other medical conditions that could affect the ability to participate in the study
Ganderton et al. 2018	Computer generated block randomization	Allocation performed by an external investigator	Participants blinded to group allocation but knew education was consistent across groups Blind assessors for the group	80%	The sham exercise group was heavier, with a higher BMI	Being postmenopausal (> 52 weeks of menstrual cessation), have reproduction of lateral hip pain in three of the five pain provocation tests and understand the English language	Hip injection in the previous 12 weeks (PRP, autologous blood injection, or CI), history of hip trauma or surgery on the affected side or any other musculoskeletal, neurologic, and cardiorespiratory conditions affecting ability to participate in the study
Mellor et al. 2018	Computer generated by independent organization	Sealed opaque envelopes, by an independent researcher	Participants blinded to study hypothesis but not to treatment Outcome assessors and statistician blinded to group allocation	80%	No difference	Age 35–70 years, lateral hip pain for more than three months, pain intensity of at least 4/10 on a numerical rating scale, clinical diagnosis of gluteal tendinopathy by a physical therapist and confirmed by MRI findings	Low back pain, intensity of sciatica or groin pain of more than 2/10 on a numerical rating scale, CI use in the previous 12 months, current physical therapy, total hip replacement, and other neurological conditions
Rompe et al. 2009	Sequentially scheduled by a secretary, indicating them for consultation A, B, and C	None	None	80%	No difference	Local tenderness to palpation of the greater trochanter area of patients with this symptom as a reason for consultation, localized pain anterior, lateral, or posterior to the greater trochanter for more than 6 months, pain when lying on the affected side, positive resisted ER test, no radiological evidence of osteoarthritis of the hip or knee joint	Acute trauma, other cause of hip pain such as sciatica, dysplasia and deformities, hip IR ≤ 20° due to pain, general myofascial tenderness to palpation, bilateral GTPS, injection in the trochanteric area during the previous 6 months, surgery of the spine and hip, acute low back pain, local infection in the hip joint, clotting disorders or anticoagulant use, vascular, neurological, or neoplastic comorbidity

*RCT* randomized clinical trial, *IR* internal rotation, *BMI* body mass index, *CI* corticosteroid injection, *ER* external rotation, *FSH* follicle-stimulating hormone, *GTPS* greater trochanteric pain syndrome, *PRP* platelet rich plasma, *ESWT* extracorporeal shock wave therapy, *NSAIDs* non-steroidal anti-inflammatory drugs, *ROM* range of motion, *MRI* magnetic resonance imaging, *MHT* transdermal hormone replacement.

### Patient characteristics

The total number of patients involved in this review was 747, with sample sizes ranging from 30 to 229 patients in the different studies (average sample size = 118.3). The mean age of the patients was 56.9 ± 5.8 years (range 47.6‒61.8) and 582 (78%) were women. The average dropout rate from studies was 10.8 ± 8.1% (range 6.9‒23.3).

### Outcome measures

Pain intensity was assessed with the NRS^37–39^. Function and disability were the outcomes that presented the greatest variability in measurement instruments, being assessed by the Hip Disability and Osteoarthritis Outcome Score (HOOS)^37,40,41^, VISA-G^37,38,40,41^, OHS^40,41^, LHPQ^38,41^, Patient Specific Functional Scale (PSFS)^38^. The perception of change was assessed using the GROC^38,39,41,42^ and the Likert Scale^39^. The EQ-5D^37,38^ and AQol^40,41^ were used to assess quality of life. The level of physical activity was assessed by the IPAQ^37^ and AAS^38^. Pain catastrophizing was assessed with the PCS^37,38^ and for strength assessments isometric torque was measured^38^. No outcome measures were found for biomechanical analyses.

### Methodological quality and certainty of evidence

The average score of the studies in the methodological quality and risk of bias assessment was 6.8 ± 1.7 points, ranging from 5 to 9 points (Table 3). Two studies presented a higher risk of bias^37,39^. The least scored topics were subject blinding (not scored by 3 studies)^37–39^, therapist blinding (not scored by 5 studies)^37–41^ and rater blindness (not scored by 2 studies)^37,39^. The analysis of the certainty of evidence in relation to the results of this review showed a range from low certainty of evidence to very low certainty of evidence (Table 4).

**Table 3:** Analysis of risk of bias using the physiotherapy evidence based database (PEDro) scale from 0 to 10.

Reference	Random allocation	Allocation concealment	Baseline comparability	Blind subjects	Blind therapists	Blind assessors	Proper follow-up	Intention to treat analysis	Comparisons between groups	Point measure and variability	Total score (0 to 10)
Cowan, 2022	Y	Y	Y	Y	N	Y	Y	Y	Y	Y	9
Clifford, 2019	Y	Y	Y	N	N	N	N	N	Y	Y	5
Ganderton, 2018	Y	Y	Y	Y	N	Y	Y	Y	Y	Y	9
Mellor, 2018	Y	Y	Y	N	N	Y	Y	Y	Y	Y	8
Rompe, 2009	N	N	Y	N	N	N	Y	Y	Y	Y	5

*Y* yes, *N* no.

**Table 4:** Analysis of the certainty of evidence using grading of recommendations assessment (GRADE) on the effects of exercise on pain intensity, function, and quality of life in gluteal tendinopathy.

Compared treatments	Summary of results	Pain intensity	Function	Quality of life
Exercise vs minimal interventions	The effect of exercise was superior to minimal interventions in the short and long term for function and was no different for quality of life in the short and long term	–	⨁⨁◯◯ Low^a^	⨁◯◯◯ Very low^a,b^
Exercise vs corticosteroid injections	The effect of exercise was not different from corticosteroid injections in the short and long term	⨁⨁◯◯ Low^b^	–	–

All outcomes were downgraded due to Inaccuracy (less than 400 participants included in the analysis).

–, did not evaluate the outcome.

^a^Downgraded to indirect evidence: sample composed of postmenopausal women (> 52 weeks of menstrual cessation). Therefore, we cannot assume external validity for any individual with gluteal tendinopathy.

^b^Downgraded to inconsistency: statistics I^2^ was greater than 50%.

### Meta-analysis

#### Effects of exercise compared to minimal interventions for function

**Figure 2:**
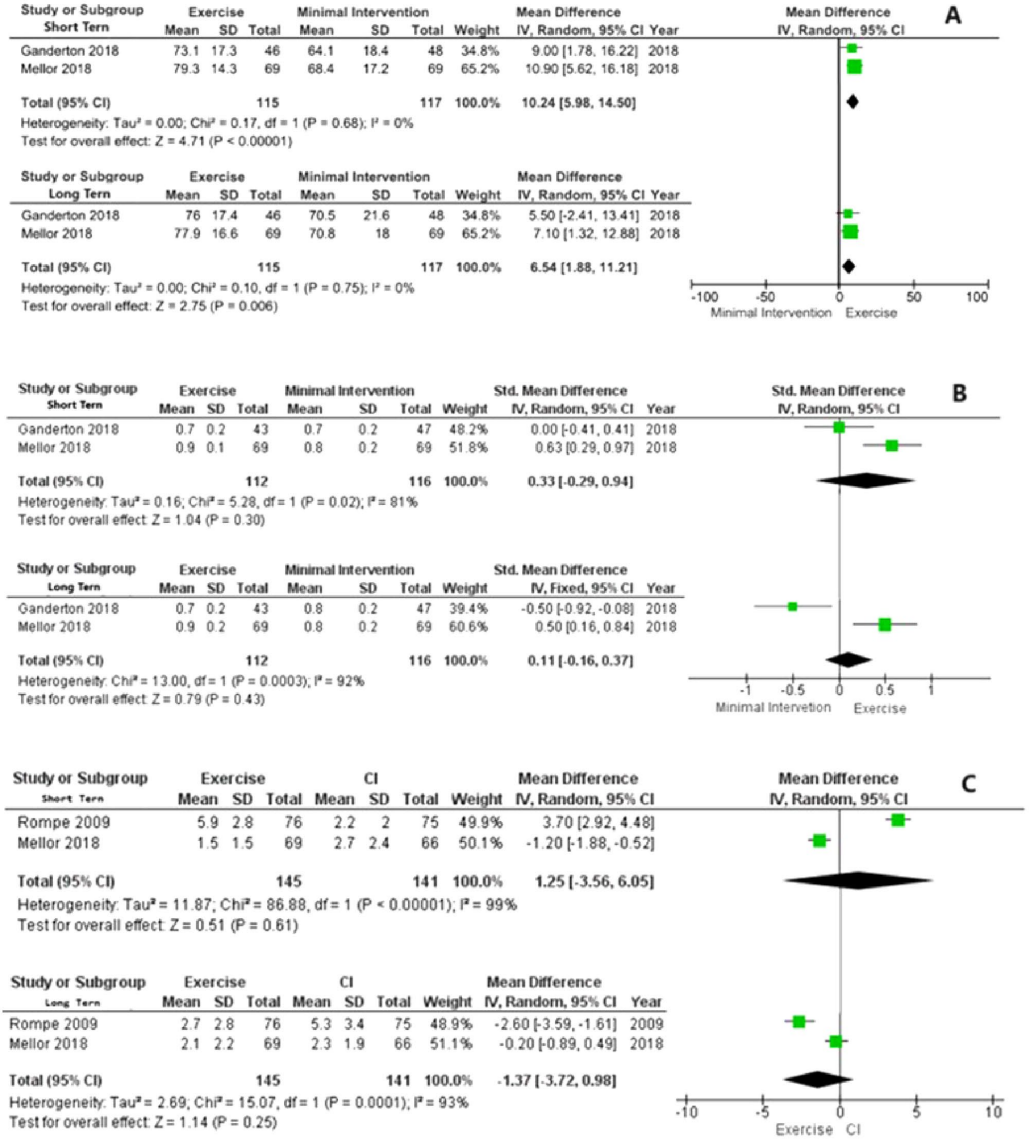
Comparison between exercise and minimal intervention for short- and long-term function (**A**) and for quality of life in the short- and long-term (**B**). Comparison between exercise and corticosteroid injections (CI) for short- and long-term pain intensity (**C**).

In the analyses for function, the meta-analysis showed that the effect of exercise was superior to minimal interventions in the short-term (MD = 10.24 [95%CI = 5.98, 14.50]; p < 0.001; n = 232) and in the long-term (MD = 6.54 [95%CI = 1.88, 11.21]; p = 0.006; n = 232) (Fig. 2A). No heterogeneity was identified in these short-term (I^2^ = 0%) and long-term (I^2^ = 0%) analyses (Fig. 2A). GRADE analysis revealed low certainty of evidence (Table 4).

#### Effects of exercise compared to minimal interventions for quality of life

In the quality of life analyses, the meta-analysis indicated that the effect of exercise was not different from minimal interventions in the short-term (SMD = 0.33 [95%CI = −0.29, 0.94]; p = 0.301; n = 232) and long-term (SMD = 0.11 [95%CI = −0.16, 0.37]; p = 0.43; n = 232) (Fig. 2B). High heterogeneity was identified in these short-term (I^2^ = 81%) and long-term (I^2^ = 92%) analyses (Fig. 2B). GRADE analysis revealed very low certainty of evidence (Table 4).

#### Effects of exercise compared to corticosteroid injections for pain intensity

In the pain intensity analyses, the meta-analysis showed that the effect of exercise was not different from corticosteroid injections in the short-term (MD = 1.25 [95%CI = −3.56, 6.05]; p = 0.61; n = 286) and long-term (MD = −1.37 [95%CI = −3.72, 0.98]; p = 0.25; n = 286) (Fig. 2C). High heterogeneity was identified in these short-term (I^2^ = 99%) and long-term (I^2^ = 93%) analyses (Fig. 2C). GRADE analysis revealed low certainty of evidence (Table 4).

#### Secondary outcomes, subgroup and sensitivity analyses

A quantitative analysis of secondary outcomes was planned; however, meta-analyses were not possible since no more than one study was found assessing these outcomes. One good quality study^38^ found that exercise had a higher treatment success rate when compared to corticosteroid injection (MD = 19.9% [95%CI = 4.7, 35.0]) and to wait-and-see (MD = 49.1% [95%CI = 34.6, 63.5]) in the short-term (8 weeks). In the long-term analysis (52 weeks), the exercise group also showed a higher treatment success rate when compared to corticosteroid injection (MD = 20.4% [95%CI = 4.9, 35.9]) and to wait-and-see (MD = 26.8% [95%CI = 11.3, 42.3]). Corticosteroid injection had a higher treatment success rate when compared to wait-and-see (MD = 29.2% [95%CI = 13.2, 45.2]) in the short-term (8 weeks) but in the long-term (52 weeks) there was no difference in treatment success rate between corticosteroid injection and wait-and-see (MD = 6.4% [95%CI =  − 10.7, 23.6])^38^.

Hip abductor strength was significantly higher after 8 weeks of an exercise intervention in relation to wait-and-see (MD = 0.1 Nm/kg [95%CI = 0.01, 0.2]) in a study with good methodological quality^38^. However, no difference was observed between corticosteroid injection and wait-and-see (MD = 0.1 Nm/kg [95%CI = −0.02, 0.2]) or between exercise and corticosteroid injection (MD = 0.02 Nm/kg [95%CI = −0.1, 0.1])^38^. With regards to pain catastrophizing, one good quality study found that exercise was superior to wait-and-see in the short-term (MD = −2.6 [95%CI = −5.0, −0.1]), however, no difference was observed between exercise and corticosteroid injections (MD = −1.7 [95%CI = −4.1, 0.7]) or between corticosteroid injections and wait-and-see (MD = −0.8 [95%CI = −3.0, 1.3]) for this outcome^38^. Adverse events associated with the interventions of the studies included in this review were uncommon and more frequently involved a short-term increase in pain (Table 1). None of the sensitivity and subgroup analyses to explore the potential impact of high risk of bias, therapy dosage, exercise type, and population characteristics were investigated due to the small number of included studies.

## Discussion

The present review investigated the effects of exercise-based interventions on pain intensity, function, and quality of life in patients with gluteal tendinopathy. The main result of this study is that exercise is superior to minimal intervention (sham exercise or wait-and-see) for function/symptom severity in patients with gluteal tendinopathy in the short- and long-term. However, no difference was observed between these interventions for short- and long-term quality of life. Similarly, the effect of exercise was no different from corticosteroid injections for pain intensity in the short- and long-term, however, exercise showed a higher treatment success rate when compared to corticosteroid infiltration both in the short- and long-term in individuals with gluteal tendinopathy. Overall, there was high heterogeneity in the studies, except for comparisons between exercise and minimal intervention. There was also low or very low certainty of evidence for these comparisons.

Management of tendinopathies generally involves exercise as the first line of treatment^15–17,19^ and exercise has been shown to improve functional outcomes in different tendinopathies^23,42,43^. Previous studies have noted that progressive exercise has produced superior results when compared to minimal interventions (i.e., wait-and-see) in terms of function in tendinopathies involving other tendons^44,45^. This finding reinforces the results found in our low heterogeneity analysis, suggesting that the natural history of the disease is not favorable for improving symptoms in individuals with tendinopathies and exercise-based interventions are important for clinical improvements.

Although some studies^40,41^ have demonstrated similar benefits comparing resistance exercise to sham exercises (exercises which do not generate tension in the gluteus medius and minimus muscles), our analyses indicate that resistance exercise yields superior results in function/severity of symptoms. However, these results did not extend to quality of life. Studies included in the quantitative and qualitative analyzes that included education on load management and avoiding tendon compression as a co-intervention^37,38,40,41^ showed favorable results in terms of clinical improvement. It is believed that activities or positions that cause prolonged or repetitive compression on tendons may worsen symptoms in patients with tendinopathy^28,46^. Therefore, it is possible that this education approach is an important aspect for the management of patients with gluteal tendinopathy. However, studies are needed to elucidate the isolated effects of education and exercise.

Furthermore, we found that the effect of exercise was similar to that of corticosteroid injections for short- and long-term pain intensity when the results of two clinical trials^38,39^ were pooled in the meta-analysis. However, this analysis presented substantial heterogeneity and low certainty of evidence. Therefore, it is unclear which of these two treatment strategies is superior in terms of short- and long-term pain reduction. One high quality study, however, showed that exercise results in a higher treatment success rate when compared to corticosteroid infiltration both in the short- and long-term in individuals with gluteal tendinopathy^38^. This result corroborates with the existing evidence that resistance exercise is more effective than passive interventions in reducing pain and improving function in tendinopathies^17,45^.

Although the use of adjunct treatments such as corticosteroid injections is common in the treatment of gluteal tendinopathy, the real benefit of this approach is not yet well understood. A review that analyzed the effect of corticosteroid injections compared to no treatment concluded that corticosteroid injections have no significant effect on reducing pain and improving function in the short- and long-term^42^. Other studies indicate that the effects of corticosteroid injections are favorable in the short-term, however its benefits seem to decrease after 3 and 6 months^38,47^.

Various exercise modalities were investigated in the studies included in our analyses, such as isometric and kinetic chain exercises, isolated isometric and isotonic exercises, strengthening exercises with progressive load, functional exercises, stretching, and home exercises. Therefore, we cannot determine whether there is a specific type of exercise that is more favorable for the treatment of gluteal tendinopathy, since regardless of the modality, they all had positive effects in the outcomes studied. A pattern that we can see comparing all the included protocols is the fact that they all followed a daily exercise regimen and were carried out for 12 weeks^37–41^. What is not clear in all studies is how the initial exercise intensity was established and how this load was monitored and progressed. Normally in clinical trials involving exercises in the management of tendinopathies, the load is recommended to be gradually increased as long as there is no significant increase in pain (3–5/10 on a numerical pain scale)^17,48^. This approach seems to be an important aspect for the evolution of exercise intensity in tendinopathy^48^.

A recent review indicates that resistance exercises for the treatment of tendinopathies should involve progressive loads, reaching high intensities to ensure a sufficient mechanical stimulus to the tendon^48^. It was also pointed out that the time for recovery from the stimuli needs to be adequate^48^, which converges with the training frequency observed in current published protocols. Recent clinical trials have concluded that different exercise modalities result in reduced symptom severity and disability in tendinopathy^49,50^. However, because exercise is a complex treatment modality to apply, as it involves several parameters that need to be adjusted, more high-quality clinical trials are necessary to define the ideal exercise dose for the treatment of gluteal tendinopathy.

The secondary outcomes qualitative analyses indicates that exercise-based interventions have positive effects in terms of hip abductor strength and pain catastrophizing in comparison to wait-and-see, based on the results of one high quality study^38^. These results are not surprising since increases in strength are expected after resistance exercises and it is plausible that with the process of gradual and progressive exposure to exercise, individuals decrease their excessive fear and catastrophizing thoughts.

This systematic review has strengths, such as the fact that it was conducted following the recommendations of the Cochrane Handbook for Systematic Reviews^26^, using the PRISMA checklist and flowchart^24^, and the GRADE approach to verify the certainty of evidence and strength of recommendations^36^. Furthermore, to our knowledge, no review has directly compared the effect of exercise to other conservative interventions for the treatment of gluteal tendinopathy. Therefore, the results of this study will help clinicians who work in the management of this condition to make assertive decisions based on evidence of high methodological rigor.

However, this review presents some limitations, especially in the development of meta-analyses. Because the outcomes of the different studies were not reported using the same measure (perception of treatment success, quality of life, level of physical activity, pain catastrophizing), it was not possible to make comparisons with these outcomes. Due to the small number of studies included, it was also impossible to group data to perform sensitivity and subgroup analyzes with the aim of exploring the potential effect of types of exercises, therapy dosage and population characteristics. Furthermore, publication bias was not assessed using funnel plots due to the limited number of studies included. Although studies indicate positive effects of exercise, the results of this review should be interpreted with caution because the certainty of the evidence ranged from low to very low.

## Conclusion

Exercise-based interventions, including progressive loading and education are superior to minimal interventions (sham exercise or wait-and-see) in terms of short- and long-term function/symptom severity in individuals with gluteal tendinopathy. Regarding pain intensity, exercise-based interventions and corticosteroid infiltrations had similar effects in this population, however, exercise showed a higher treatment success rate when compared to corticosteroid infiltration both in the short- and long-term in individuals with gluteal tendinopathy. The certainty of the evidence varied from low to very low, therefore, large high-quality, randomized controlled trials are recommended.

### Supplementary Information

Supplementary Information.

## Data Availability

The datasets used and/or analysed during the current study available from the corresponding author on reasonable request.
