# Supplementary material for: Effects of exercise-based interventions on gluteal tendinopathy. Systematic review with meta-analysis
Source: Sci Rep. 2024 Feb 9;14:3343. doi: 10.1038/s41598-024-53283-x (PMC10858207; doi:10.1038/s41598-024-53283-x)
Supplement: Supplementary file 1 — Supplementary Information. [file 41598_2024_53283_MOESM1_ESM.docx]

**SUPPLEMENTARY INFORMATION**

**APPENDIX A**

**Search Strategy**

**MEDLINE (PUBMED)**

greater trochan* pain syndrome OR trochan* bursitis OR lateral hip pain OR lateral hip tend* OR glute* tend* OR glute* mini* tend* OR glute* medi* tend* OR hip AND enthes* OR hip AND bursitis OR iliotibial band OR enthes* AND trochanteric bursitis OR glute* bursitis AND eccentric OR concentric OR isometric OR train* OR exercis* OR rehab* OR load* OR resistance OR physiotherapy OR physical* OR therapy OR strength* OR isotonic OR motion OR endur* OR weight* OR runn* OR treadmill* OR swim* OR bicycl* OR (cycle* OR cycling) OR walk* AND randomized controlled trial OR “controlled clinical trial OR randomized OR placebo OR clinical trials as topic OR randomly OR trial NOT (animals OR nonhuman)

**EMBASE**

('greater trochan* pain syndrome' OR 'trochan* bursitis' OR 'lateral hip pain' OR 'lateral hip tend*' OR 'glute* tend*' OR 'glute* mini* tend*' OR 'glute* medi* tend*' OR (hip AND enthes*) OR (hip AND bursitis) OR 'iliotibial band' OR (enthes* AND 'trochanteric bursitis') OR 'glut* bursitis') AND (eccentric OR concentric OR isometric OR train* OR exercis* OR rehab* OR load* OR resistance OR physiotherapy OR physical* OR therapy OR strength* OR isotonic OR motion OR endur* OR weight* OR runn* OR treadmill* OR swim* OR bicycl* OR cycle* OR cycling OR walk*) AND ('randomized controlled trial' OR 'controlled clinical trial' OR randomized OR placebo OR 'clinical trials as topic' OR randomly OR trial) NOT (animals OR nonhuman)

**CINAHL (EBSCO HOST)**

(((((("greater trochan* pain syndrome") OR ("trochan* bursitis") OR ("lateral hip pain") OR "S4" OR ("glute* tend*") OR ("glute* mini* tend*") OR ("glute* medi* tend*") OR ((hip) AND (enthes*)) OR ((hip) AND (bursitis)) OR ("iliotibial band") OR ((enthes*) AND ("trochanteric bursitis")) OR (glute* bursitis)) AND (eccentric OR concentric OR isometric OR train* OR exercis* OR rehab* OR load* OR resistance OR physiotherapy OR physical* OR therapy OR strength* OR isotonic OR motion OR endur* OR weight* OR runn* OR treadmill* OR swim* OR bicycl* OR ((cycle*) OR (cycling)) OR walk*))) AND ((("randomized controlled trial") OR ("controlled clinical trial") OR randomized OR placebo OR ("clinical trials as topic") OR trial OR randomly)))) NOT (((animals) OR (nonhuman)))

**PEDro**

Abstract & Title: glut* tend* exercis*

Therapy: not applicable

Problem: not applicable

Body Part: not applicable

Subdiscipline: not applicable

Topic: not applicable

Method: not applicable

Author/Association: not applicable

Title Only: not applicable

Source: not applicable

Published Since: not applicable

New records added since: not applicable

Score of at least: not applicable

- Keywords combined with OR operator

**COCHRANE**

#1 Greater trochan* pain syndrome

#2 trochan* bursitis

#3 Lateral hip pain

#4 Lateral hip tend*

#5 Glute* tend*

#6 Glute* mini* tend*

#7 Glute* medi* tend*

#8 HIP

#9 enthes*

#10 #8 and #9

#11 bursitis

#12 #8 and #11

#13 Iliotibial band

#14 trochanteric bursitis

#15 #9 and #14

#16 glut* bursitis

#17 #1OR#2OR#3OR#4OR#5OR#6OR#7OR#10OR#12OR#13OR#15OR#16

#18 eccentric

#19 concentric

#20 isometric

#21 train*

#22 exercis*

#23 Rehab*

#24 load*

#25 resistance

#26 physiotherapy

#27 physical*

#28 therapy

#29 strength*

#30 isotonic

#31 motion

#32 endur*

#33 Runn*

#34 treadmill*

#35 swim*

#36 bicycl*

#37 cycle*

#38 cycling

#39 #37 or #38

#40 walk*

#41 #18OR#19OR#20OR#21OR#22OR#23OR#24OR#25OR#26OR#27OR#28OR#29OR#30OR#31OR#32OR#33OR#34OR#35OR#36OR#39OR#40

#42 randomized controlled trial

#43 controlled clinical trial

#44 randomized

#45 placebo

#46 clinical trials as topic

#47 randomly

#48 trial

#49 #42OR#43OR#44OR#45OR#46OR#47OR#48

#50 animals

#51 nonhuman

#52 #50 or #51

#53 #17 AND #41 AND #49

#54 #53 NOT #52
